# Inhibition of cancer cell proliferation and apoptosis-inducing activity of fungal taxol and its precursor baccatin III purified from endophytic *Fusarium solani*

**DOI:** 10.1186/1475-2867-13-105

**Published:** 2013-10-23

**Authors:** Balabhadrapatruni VSK Chakravarthi, Ramanathan Sujay, Gini C Kuriakose, Anjali A Karande, Chelliah Jayabaskaran

**Affiliations:** 1 Department of Biochemistry, Indian Institute of Science, Bangalore 560012, India; 2 Present Address: Michigan Center for Translational Pathology, Ann Arbor 48109-0602, MI, USA; 3 Present Address: Department of Pathology, University of Michigan, Ann Arbor 48109-0602, MI, USA

**Keywords:** Taxol, Baccatin III, Apoptosis, Endophyte, Anticancer, Caspase, Jurkat, Mitochondria

## Abstract

**Background:**

Taxol (generic name paclitaxel), a plant-derived antineoplastic agent, used widely against breast, ovarian and lung cancer, was originally isolated from the bark of the Pacific yew, *Taxus brevifolia*. The limited supply of the drug has prompted efforts to find alternative sources, such as chemical synthesis, tissue and cell cultures of the *Taxus* species both of which are expensive and yield low levels. Fermentation processes with microorganisms would be the methods of choice to lower the costs and increase yields. Previously we have reported that *F. solani* isolated from *T. celebica* produced taxol and its precursor baccatin III in liquid grown cultures J Biosci 33:259-67, 2008. This study was performed to evaluate the inhibition of proliferation and induction of apoptosis of cancer cell lines by the fungal taxol and fungal baccatin III of *F. solani* isolated from *T. celebica*.

**Methods:**

Cell lines such as HeLa, HepG2, Jurkat, Ovcar3 and T47D were cultured individually and treated with fungal taxol, baccatin III with or without caspase inhibitors according to experimental requirements. Their efficacy on apoptotic induction was examined.

**Results:**

Both fungal taxol and baccatin III inhibited cell proliferation of a number of cancer cell lines with IC_50_ ranging from 0.005 to 0.2 μM for fungal taxol and 2 to 5 μM for fungal baccatin III. They also induced apoptosis in JR4-Jurkat cells with a possible involvement of anti-apoptotic Bcl2 and loss in mitochondrial membrane potential, and was unaffected by inhibitors of caspase-9,-2 or -3 but was prevented in presence of caspase-10 inhibitor. DNA fragmentation was also observed in cells treated with fungal taxol and baccatin III.

**Conclusions:**

The cytotoxic activity exhibited by fungal taxol and baccatin III involves the same mechanism, dependent on caspase-10 and membrane potential loss of mitochondria, with taxol having far greater cytotoxic potential.

## Background

Taxol (Paclitaxel) is a complex diterpenoid compound originally isolated from the bark of Pacific yew tree, *Taxus brevifolia*[1]. It is the drug of choice with significant antitumor activity towards ovarian, breast and lung cancers [2,3]. An exciting development announced in 1993 was that taxol could be produced by the fungus *Taxomyces andreanae*[4]. Several taxol-producing endophytic fungi have been identified since, such as *Taxomyces andreanae, Taxodium disticum, Tubercularia* sp*., Pestalotiopsis microspora, Alternaria* sp.*, Fusarium maire* and *Periconia* sp [5-10]. It is clear that plants and endophytic fungi produce similar secondary metabolites through mutual symbiosis. Recently, it was reported that plants other than *Taxus* species also harbor endophytic fungi that produce taxol. For example, the endophyte *Periconia* sp from *Torreya grandifolia* (a relative of yew that does not synthesize taxol) [7], *Pestalotiopsis guepinii* from *Wollemia nobilis*[11], and *Bartalinia robilldoides* Tassi from the medicinal plant *Aegle marmelos* Cornea ex Roxb of India [12] have been shown to produce taxol in culture.

Ample evidence exists to show the induction of apoptosis by taxol treatment in diverse cancer cells, including breast cancer, glioblastoma, hepatoma and ovarian cancer. Taxol is known to trigger apoptosis by both caspase-dependent [13-19] and caspase-independent pathways [20-23]. One of the main supporting observations for the latter is the failure of the pancaspase inhibitor (Z-VAD-FMK) to rescue cells from taxol-induced apoptosis [20,22]. It is shown that caspase-3 and -8 (death-receptor independent) are involved in taxol-induced apoptosis of Burkitt’s lymphoma BJAB cells through the mitochondrial amplification loop [24].

Earlier, we isolated a taxol-producing endophyte *F. solani* IISc CJB-1, standardized the growth conditions of this fungus and purified taxol [25]. In the preliminary characterization studies, we demonstrated that the fungal taxol triggered apoptosis in the human Jurkat T cell line [25]. Subsequently, baccatin III was purified from the fungus (Chakravarthi and Jayabaskaran, unpublished data). In the current study, we characterize and compare the antiproliferative and apoptosis inducing activity of the fungal taxol and baccatin III in other cell lines, as well as delineate the pathway of trigger of apoptosis.

## Methods

### Chemicals and reagents

Baccatin III, Dimethyl sulfoxide (DMSO), Hoechst 33258, Paclitaxel (Taxol), propidium Iodide (PI), Proteinase K and RNase A were purchased from Sigma-Aldrich. Pancaspase inhibitor, caspase-2 inhibitor, caspase-3 inhibitor, caspase -9 inhibitor and caspase-10 inhibitor were obtained from R&D systems Inc. (Minneapolis, MN) and Calbiochem. Dulbecco’s modified Eagle medium (DMEM), RPMI-1640 medium and fetal bovine serum (FBS) were purchased from GIBCO. JC-1 (5, 5′, 6, 6′-tetrachloro-1, 1′, 3, 3′-tetraethylbenzimidazolyl carbocyanine iodide) dye was purchased from Molecular probes (Eugene, OR, USA). All other reagents and compounds were of analytical grade.

### Isolation of taxol and baccatin III from *F. solani*

As described earlier [25], taxol and baccatin III were isolated from *F. solani*. Briefly, the fungus was grown in 500 ml of potato dextrose liquid medium in 2 l Erlenmeyer flasks at 25°C in the dark in stationary cultures. After 21 days, 1 l of culture (medium plus mycelium) was extracted twice with an equal volume of methylene chloride [8]. The obtained crude extract was subjected to thin-layer chromatographic analysis using solvent system, chloroform:methanol (9.2:0.8; v/v). After chromatography, the area with silica gel on the plates containing putative taxol and baccatin III was scraped at the appropriate relative front (Rf) and exhaustively eluted with methanol and further separated by high performance liquid chromatography (HPLC) using Kromasil C18-column (250 × 4.6 mm) at 227 nm. Authentic taxol and baccatin III standards were used as reference.

### Cell lines and culture conditions

HeLa (human cervical carcinoma cell line), HepG2 (human liver carcinoma cell line), Jurkat-JR4 (human leukemia T cell line), Ovcar-3 (human ovarian carcinoma cell line), T47D (human ductal breast epithelial tumor cell line), Jurkat-JR16 (Jurkat cells overexpressing Bcl2) and caspase-8 deficient Jurkat cells were used for the experiments. The Jurkat cell lines were grown in RPMI-1640 medium. HepG2, HeLa, Ovcar-3 and T47D cells were cultured in DMEM. All culture media were supplemented with 10% fetal bovine serum (FBS), 100 iu ml^-1^ penicillin and 100 μg ml^-1^ streptomycin. Cell lines were grown in a humidified 5% CO_2_ environment at 37°C and were passaged every 3–4 days. Stock solutions of paclitaxel, baccatin III, fungal taxol and fungal baccatin III dissolved in DMSO were stored at -80°C. Stocks were diluted in culture medium at the required concentration at the time of treatment.

### Analysis and quantification of apoptosis

Analysis of hypodiploid cells were performed using Propidium Iodide (PI) staining [26]. Flow cytometric analysis (FACScan) of the cell lines was performed after treatment with taxol and baccatin III. Cells (0.5 × 10^6^) treated with different concentrations of standards or fungal taxol and baccatin III in 500 μl of medium for various time intervals were harvested and washed once with 0.2% BSA containing PBS (50 mM phosphate buffer, pH 7.2 containing 0.85% NaCl) and fixed in 70% ethanol for 1 h at -20°C. The cells were then centrifuged at 1000 × *g* and suspended in staining solution containing 50 μg/ml PI, 50 μg/ml RNase A and 100 μM EDTA in PBS for 1 h at 42°C. Analysis was carried out using a flow cytometer. Cell cycle distribution is presented as the number of cells versus the amount of DNA, and the extent of apoptosis was determined by counting cells of DNA content within the subG1 peak.

### Effect of caspases on fungal taxol and baccatin III induced apoptosis

In order to find out the involvement of caspases in the fungal taxol and baccatin III induced apoptotic pathway, caspase inhibitors were employed. Jurkat cells (0.25 × 10^6^) in 250 μl of RPMI supplemented with 10% FBS were first pretreated with 25, 50 and 100 μM of cell permeable Z-VAD-FMK (inhibitor of all caspases) or Z-LEHD-FMK (caspase 9 inhibitor) or Z-DEVD-FMK (caspase 3 inhibitor) or Z-AEVD-FMK (caspase-10 inhibitor) or Z-VDVAD-FMK (caspase-2 inhibitor) for 1 h. The cells were then cultured for 24 and 48 h with 6 nM of fungal taxol (T_FUNG_) or 3.5 μM of fungal baccatin III (B_FUNG_). The cells were processed for PI staining and subjected to FACScan analysis as described above.

### Determination of the mitochondrial membrane potential (JC-1 Assay)

The change in mitochondrial membrane potential or MMP (ΔΨ_m_) was measured using the potentiometric dye JC-1 as described earlier [27]. The assay was carried out in 24-well plates. Cells were treated with fungal taxol (6 nM) or fungal baccatin III (3.5 μM) for 6, 12, 24 and 36 h. The cells were then incubated with 2.5 μg ml^-1^ of JC-1 dye for 15 min at 37°C, washed once with ice-cold PBS containing 2% (v/v) FBS, resuspended in the same and analyzed immediately by flow cytometry. JC-1 monomers emit at 530 nm (FL-1 channel- green fluorescence) and J-aggregates emit at 590 nm (FL-2 channel- red fluorescence). 2, 4-Dinitrophenol (2,4-DNP) is used as the positive control to set the gates along with the untreated cells as the negative control. The percentage of MMP (MFI_590nm_/MFI_525nm_) was plotted against time upon fungal taxol or baccatin III treatment. Data analysis was carried out using CellQuest Pro software.

### Determination of nuclear morphology

The changes in chromatin organization upon treatment with fungal taxol or baccatin III was determined microscopically by staining either with Hoechst 33258 or acridine orange-ethidium bromide (AO-EB) dual stain [28]. After overnight adherence on cover slips (in case of HeLa cells), the cells were incubated with fungal taxol (0.1 μM for both JR4-Jurkat and HeLa cells) or baccatin III (3.5 μM for JR4-Jurkat and 3 μM for HeLa cells) for 12 h. The cells were then fixed with 3.7% (v/v) paraformaldehyde, permeabilized with 0.1% Triton X-100 and stained with Hoechst 33258 (1 mg ml^-1^ in PBS) [29]. After washing twice with PBS, cells were examined by fluorescence microscopy (360/40 nm excitation and 460/50 nm emission filters). The apoptotic cells were identified by the presence of highly condensed chromatin or fragmented nuclei. For AO/EB staining [30], after treatment with indicated concentrations of taxol or baccatin III for 12 h, the cells were incubated with 3 μl of RNase A (10 mg ml^-1^) at 37°C for 30 min. After washing twice with PBS, the cells were fixed with 3.7% (v/v) paraformaldehyde for 10 min at room temperature. Then the cells were stained with an AO/EB mixture for 15 min and washed with PBS, the cells were observed under fluorescence microscope at 10× magnification using 485 nm excitation and 535 nm emission filter sets.

### DNA fragmentation analysis

DNA fragmentation was studied as described earlier [31]. Jurkat cells (3 × 10^6^) were treated with fungal taxol (6 nM) or baccatin III (3.5 μM), whereas HeLa cells, after overnight adherence were treated with fungal taxol (6 nM) or baccatin III (3 μM), for 36 h. After treatment, the cells were harvested and washed with 1 ml of PBS, resuspended in 100 μl of PBS and fixed in 70% chilled ethanol overnight. The cells were spun down at 1000 × g and resuspended in 40 μl of phosphate-citrate (PC) buffer consisting of 192 parts of 0.2 M Na_2_HPO_4_ and 8 parts of 0.1 M of citric acid (pH 7.8), at RT for 30 min. After centrifugation at 1000 × g at RT for 5 min, the supernatant was transferred to fresh tubes and concentrated by vacuum in SpeedVac concentrator (Savant Instruments Inc., Farmingdale, NY). 3 μl of 0.25% Nonidet-40 (Sigma Chemicals Co., USA) in distilled water was then added to the tubes, followed by 3 μl of a solution of RNase A (Sigma Aldrich Co., USA). After incubation for 30 min at 37°C, 3 μl of a proteinase K (Sigma Aldrich Co.,USA) was added and incubated for additional 30 min at 37°C. Gel loading buffer (0.25% bromophenol blue, 0.25% xylene cyanol, 30% glycerol) was the added and the entire content of the tube was transferred to 1.2% agarose gel and electrophoresed at 2 V/cm for 16 h. The DNA present in the gels was visualized under UV light after staining with ethidium bromide (5 μg ml^-1^).

### Statistical analysis

Statistical analysis was performed using GraphPad Prism software 5.0 (GraphPad Software, La Jolla, CA, USA). Student’s t-test was used to analyze the data. Values of p <0.05 or less were considered statistically significant.

## Results

### Induction of apoptosis by fungal taxol and baccatin III in Jurkat cells

**Figure 1:**
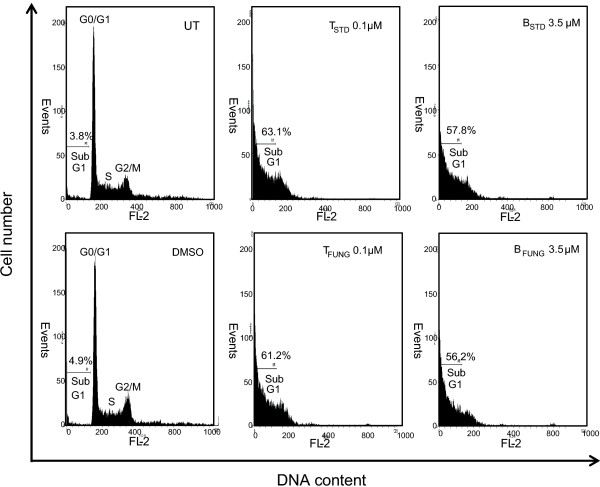
**Taxol- and baccatin III-induced apoptosis in Jurkat cells assayed with PI staining followed by flow cytometry.** Cells (0.25 × 10^6^) were treated with indicated concentrations of taxol or baccatin III for 48 h, and analyzed by PI staining followed by flow cytometry to determine the hypodiploid DNA (fragmented DNA) proportions. 1 × 10^5^ cells were analyzed. The percentage of hypodiploid cells (sub G1 peak) was calculated on the basis of the respective histograms. UT, Untreated cells; DMSO, Dimethylsulfoxide; T_STD_, standard taxol; T_FUNG_, fungal taxol; B_STD_, standard baccatin III; B_FUNG_, fungal baccatin III.

Interference of the mitotic spindle apparatus by microtubule-stabilizing drugs would be expected to have an effect on the cell cycle distribution. To determine whether taxol and its precursor would have any such effect, JR4-Jurkat cells were treated for 48 h with 0.1 μM fungal taxol and 3.5 μM baccatin III, subjected to PI staining and the DNA content of the cells measured by flow cytometry. Flow cytometry analysis showed that while untreated and vehicle (DMSO) treated Jurkat cells were predominantly in the G1 phase of the cell cycle (Figure 1), significant changes were observed with fungal taxol and baccatin III-treated cells. Upon treatment, the percentage of G1 and G2/M cells decreased and the percentage of sub G1 cells increased considerably (Figure 1), suggesting initiation of apoptosis process in the cells.

### Induction of apoptosis by taxol and baccatin III in cells

**Figure 2:**
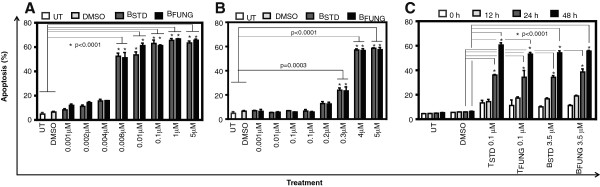
**Fungal taxol and baccatin III exhibit concentration- and time-dependent apoptosis in JR4-Jurkat cells.** Cells (0.25 × 10^6^) were treated with fungal taxol **(A)** or baccatin III **(B)** at indicated concentrations for 48 h. A time-course experiment of fungal taxol- and baccatin III-induced apoptosis **(C)**. Other details are as described in legend to Figure 1. The percentage of apoptotic cells (sub G1 peak) was calculated on the basis of respective histograms.

We observed a clear dose- and time- dependent induction of apoptosis by taxol and baccatin III in cells. The maximal increase in the frequency of apoptotic cells was observed after 48 h of incubation with 0.1 μM fungal taxol (68%) (Figure 2A and 2C), while the maximal induction of apoptosis by fungal baccatin III was obtained in 48 h at a concentration of 5 μM (65%) (Figure 2B and 2C).

Later the effect of induction of apoptosis by fungal taxol and baccatin III was analyzed in adherent cell lines. HepG2, HeLa, Ovcar3 and T47D cells treated with fungal taxol and baccatin III showed results similar to that obtained with the Jurkat cells. Time- and concentration-dependent effect of fungal taxol and baccatin III on apoptosis induction in the four different adherent cell lines was observed, though the IC_50_ concentrations differed. IC_50_ values of apoptosis were calculated from all the five different cell lines that were induced by fungal taxol and baccatin III (Table 1). Both the compounds were active in all the cancer cell lines we tested, with IC_50_ ranging from 0.005 to 0.2 μM for fungal taxol and 2–5 μM for fungal baccatin III. These results indicate that both fungal taxol and baccatin III have potent apoptosis inducing activity.

**Table 1:** Comparative analysis of apoptosis in five different cancer cell lines assayed with PI staining followed by flow cytometry

**Treatment**	**IC**_**50 **_**(μM) for apoptosis**				
	**JR4-Jurkat**	**HepG2**	**HeLa**	**Ovcar3**	**T47D**
T_FUNG_	0.006 ± 0.0003	0.1 ± 0.02	0.008 ± 0.001	0.2 ± 0.05	0.005 ± 0.001
B_FUNG_	3.5 ± 0.05	3 ± 0.1	4 ± 0.2	5 ± 0.3	2 ± 0.3

JR4-Jurkat, HepG2, HeLa, Ovcar3 or T47D cell lines were treated with either fungal taxol (T_FUNG_) (0.001-5 μM) or fungal baccatin III (B_FUNG_) (0.001-8 μM) for 48 h prior to DNA histogram analysis by flow cytometry. The IC_50_ shown were calculated using values derived from the respective histograms. The results show the mean ± SD of three independent experiments.

### Fungal taxol- and baccatin III- induced reduction of mitochondrial membrane potential in JR4 Jurkat cells

**Figure 3:**
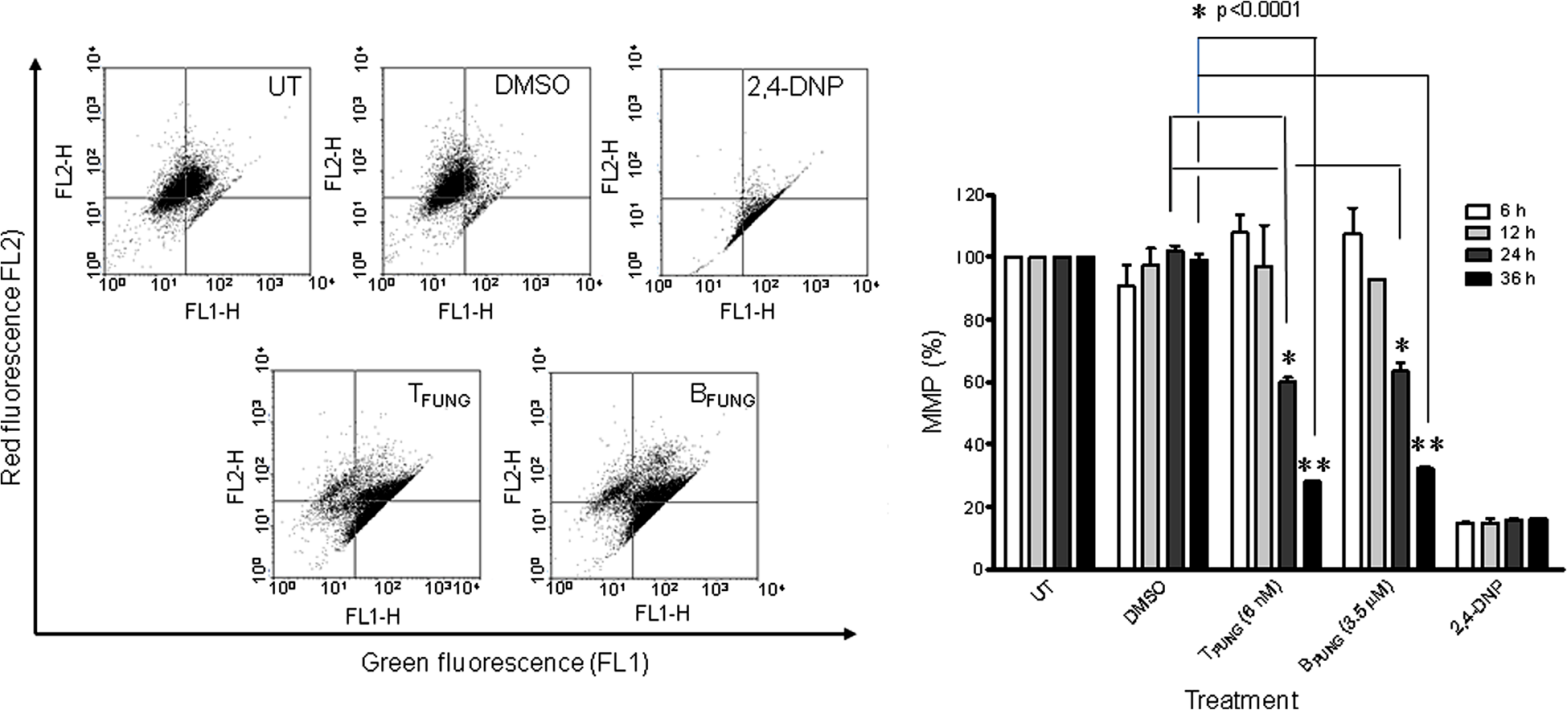
**Fungal taxol- and baccatin III- induced loss of mitochondrial membrane potential (ΔΨ**_**m**_**) in JR4-Jurkat cells determined by JC-1 staining and flow cytometry.** Jurkat cells were incubated with either fungal taxol (6 nM) or baccatin III (3.5 μM) for indicated time periods. Mitochondrial membrane potential was analyzed using JC-1 staining. Results are given in percent of 10,000 events counted in FACS analysis. In dot plots of JR4-Jurkat cells, lower right and left quadrant (FL-1) represent the percentage of apoptotic cells that emit only green fluorescence while upper right and left quadrant show the healthy population. The percentage of cells having ΔΨ_m_ was calculated taking the ratio of red to green fluoresence against time. 2,4-DNP was used as positive control. Data are expressed as mean ± SD from three independent experiments.

Disturbance in the mitochondrial membrane potential is an early event in the process of apoptosis and can be studied using the cationic carbocyanine dye JC-1 as a fluorescent marker for assessing the loss in mitochondrial membrane potential. The distinctive feature of JC-1 is its potential sensitive emission color shift resulting in a decrease of the red/green fluorescence intensity ratio in response to mitochondrial depolarization. To characterize the effect of fungal taxol and baccatin III on mitochondrial apoptotic pathway, we measured the mitochondrial membrane potential (Δψ_m_) in Jurkat cells on staining with JC-1. The depolarization of mitochondrial membrane potential increased with time upon incubation with the indicated concentrations of fungal taxol and baccatin III (Figure 3). In contrast, control and vehicle-treated samples did not exhibit significant loss in mitochondrial membrane potential. Thus, it can be concluded that activation of the mitochondrial apoptotic machinery occurs in Jurkat cells upon fungal taxol and baccatin III exposure.

### Role of caspases in fungal taxol- and baccatin III-induced apoptosis

**Figure 4:**
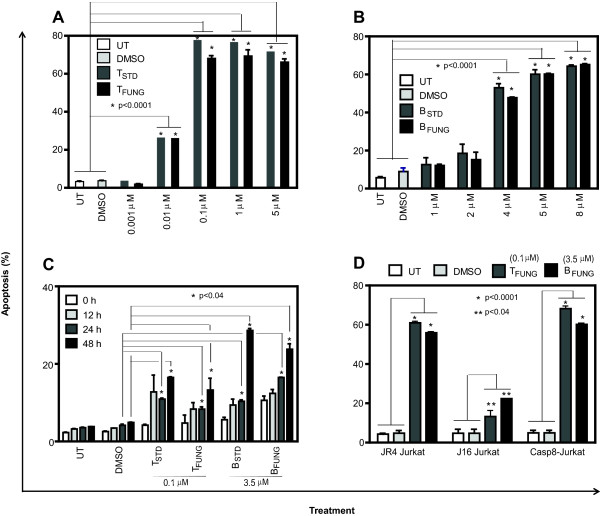
**Effect of fungal taxol and baccatin III on apoptosis.** Caspase-8-deficient Jurkat **(A and B)** and anti-apoptotic protein Bcl2-overexpressing J16 Jurkat cells **(C)** (0.25 × 10^6^) were treated with fungal taxol or baccatin III at indicated concentrations for 48 h or indicated time periods and assayed with PI staining followed by flow cytometry. The percentage of apoptotic cells (sub G1 peak) was quantified and plotted. **(D)** Relative levels of fungal taxol- and baccatin III- induced apoptosis in the three cell lines after 48 h.

It is well known that a family of cysteinyl proteases, called caspases, is involved in apoptotic cell death. To check the involvement of caspase-8 of extrinsic pathway in fungal taxol or baccatin III induced apoptosis, caspase-8-deficient Jurkat cells were treated with these compounds. Both compounds induced apoptosis in 60- 80% of cells after 48 h of treatment (Figure 4A and 4B), suggesting that caspase 8 may not be involved in taxol or baccatin III-induced apoptosis of Jurkat cells.

We then tested whether fungal taxol or baccatin III could induce apoptosis in J16-Jurkat cells that over-express the anti-apoptotic protein Bcl-2. Fungal taxol or baccatin III did not induce significant apoptosis in J16-Jurkat cells even after 48 h, which suggests that Bcl-2 overexpression rescues taxol and baccatin III- induced apoptosis (Figure 4C). A comparison of percentage of apoptosis induced by fungal taxol and baccatin III in JR4-Jurkat, J16-Jurkat and caspase-8 deficient Jurkat cells is also shown (Figure 4D). Both the compounds induced 55–60% apoptosis in JR4-Jurkat cells, 60–70% apoptosis in caspase-8 deficient Jurkat cells, while no significant apoptosis was observed in J16-Jurkat cells. This confirms the involvement of intrinsic mitochondrial pathway of apoptosis.

**Figure 5:**
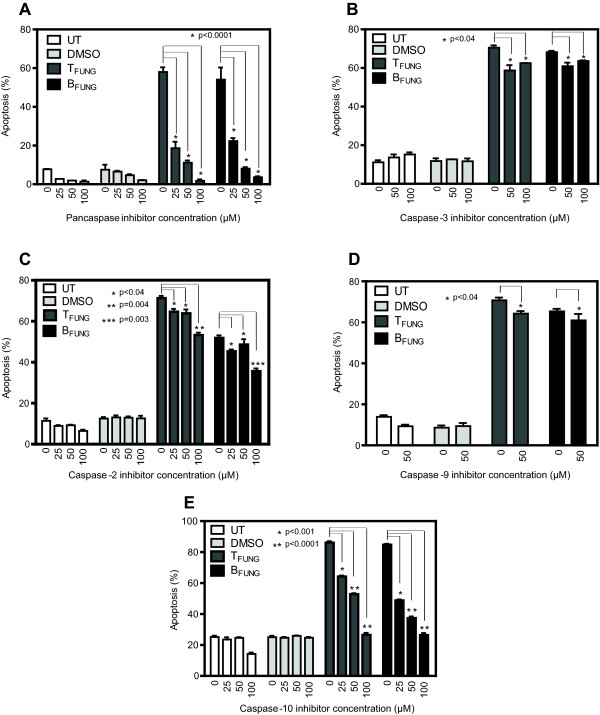
**Effect of caspase inhibitors on fungal taxol-/baccatin III- induced apoptosis.** JR4-Jurkat cells (0.25 × 10^6^) were treated with 6 nM fungal taxol (T_FUNG_) or 3.5 μM fungal baccatin III (B_FUNG_) in the presence or absence of indicated concentrations of inhibitors of **(A)** pancaspase (Z-VAD-FMK), **(B)** caspase-3 (Z-DEVD-FMK), **(C)** caspase-2 (Z-VDVAD-FMK), **(D)** caspase-9 (Z-LEHD-FMK) or **(E)** caspase-10 (Z-AEVD-FMK) for 48 h and assayed with PI staining followed by flow cytometry. The percentage of hypodiploid cells (sub-G1 peak) was calculated on the basis of the respective histograms. Data is expressed as mean ± SD from three independent experiments.

To determine which of the caspases are involved in the taxol and baccatin-induced apoptosis, the effect of specific inhibitors of these enzymes were examined using Jurkat cells. Cells were pre-treated for 1 h with pan-caspase inhibitor (Z-VAD-FMK), caspase-3 inhibitor (Z-DEVD-FMK), caspase-2 inhibitor (Z-VDVAD-FMK), caspase-9 inhibitor (Z-LEHD-FMK) or caspase-10 inhibitor (Z-AEVD-FMK) at various concentrations and then treated with either fungal taxol or baccatin III for 24 or 48 h in the continued presence of the respective caspase inhibitor. The cells were then stained with PI and subjected to FACS analysis. The pan-caspase inhibitor at 100 μM concentration showed complete rescue of Jurkat cells from fungal taxol- and baccatin III-induced apoptosis (Figure 5A). None of the inhibitors for caspase-3 (Figure 5B), caspase-2 (up to 50 μM; Figure 5C) or caspase-9 (Figure 5D) could siginificantly rescued Jurkat cells from apoptosis which indicated that these caspases are not involved in fungal taxol- and baccatin III-induced apoptotic mechanism. But we observed a siginificant rescue of these cells at 100 μM of caspase-2 inhibitor treatment (Figure 5C). Interestingly, caspase-10 inhibitor exhibited complete rescue of JR4-Jurkat cells from apoptosis (Figure 5E). This establishes both fungal taxol and baccatin III as potent inducers of apoptosis through the activation of caspase-10 in JR4-Jurkat cells and, furthermore, caspase-2 may be involved in fungal taxol and baccatin III-mediated apoptosis of JR4-Jurkat cells.

### Fungal taxol and baccatin III induce changes in nuclear morphology in JR4-Jurkat and HeLa cells

**Figure 6:**
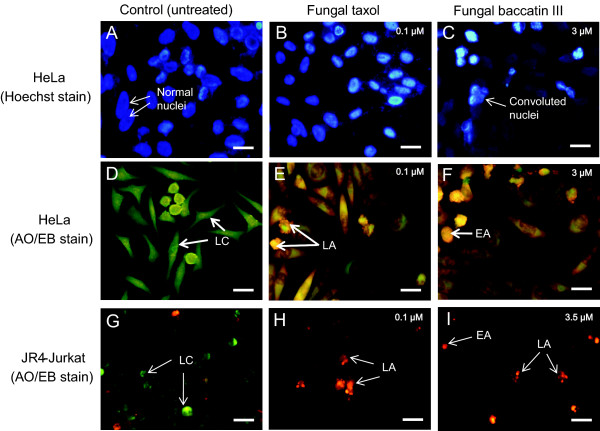
**Apoptotic effect of fungal taxol and baccatin III as determined by Hoechst or Acridine orange-ethidium bromide dual staining.** HeLa and JR4- Jurkat cells were cultured on sterile coverslips and treated with indicated concentrations of fungal taxol or baccatin III for 12 h following which the cells were stained with either Hoechst 33258 **(A-C)** or Acridine orange-Ethidium bromide **(D-F, G-I)** and viewed under a fluorescence microscope. Arrows indicate cells in early apoptosis (EA), late apoptosis (LA), live cells (LC) and convoluted nuclei. Scale bar represents 20 μm.

Changes in cell nuclear morphology, such as condensed and fragmented nuclei are considered late events of apoptosis. In order to identify the changes in cell nuclei in JR4-Jurkat and HeLa cells upon treatment with indicated concentrations of fungal taxol and baccatin III, cells were stained with Hoechst or AO-EB and visualized by fluorescence microscopy. Our data reveal that both fungal taxol and baccatin III induce chromatin aggregation and nuclear condensation in JR4-Jurkat and HeLa cells (Figure 6A-I). The control cells that stained evenly with Hoechst were also found to stain lightly and evenly with AO but stained negative for EB, suggesting the presence of live cells. On the other hand, HeLa cells after 12 h of treatment, exhibited a condensed orange nucleus, whereas the necrotic cells display a structurally intact nucleus with an evenly distributed orange staining (Figure 6D-F).

### Fungal taxol and baccatin III induce DNA fragmentation in both JR4-Jurkat and HeLa cells

**Figure 7:**
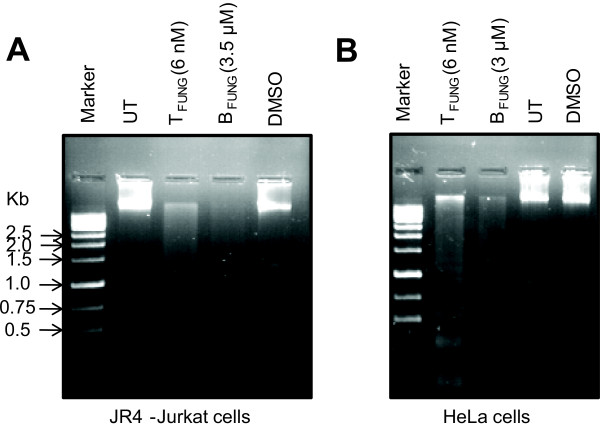
**DNA fragmentation of JR4-Jurkat and HeLa cells induced upon treatment with fungal taxol and baccatin III. (A)** JR4-Jurkat and **(B)** HeLa cells (3 × 10^6^) were treated independently with fungal taxol, baccatin III or DMSO at indicated concentrations for 36 h. After extraction, DNA was electrophoresed in a 1.2% agarose gel, stained with ethidium bromide and photographed under UV illumination.

The fragmentation of nuclear DNA is one of the hallmarks of apoptosis. It is known that DNA fragmentation is carried out by the caspase activated DNase (CAD). Activation of CAD leads to cleavage of nuclear DNA into multiples of ~200 bp oligonucleosomal size fragments. To confirm the induction of apoptosis, JR4-Jurkat and HeLa cells were treated with fungal taxol or baccatin III. Low molecular weight DNA isolated from these cells was analyzed in 1.2% agarose gels. DNA ladder formation is observed upon taxol or baccatin III treatment in JR4-Jurkat and HeLa cells, while there is no DNA fragmentation seen in untreated and DMSO treated cells (Figure 7). This confirmed that both fungal taxol and baccatin III can induce apoptosis in JR4-Jurkat or HeLa cells.

## Discussion

Taxol is a potent chemotherapeutic agent that induces apoptosis in a variety of cancer cells including ovarian [32], endometrial [33], lung [34], prostate [35], colorectal [36], thyroid [37], acute myeloid leukemia [38] and breast [39] cancer cells. It was of interest to investigate whether baccatin III, the biosynthetic precursor of taxol, functions by the same mechanism as taxol. This is the first report on the apoptotic mechanisms involved in fungal baccatin III-induced cytotoxicity in cancer cells. Comparison of cell cycle analysis of Jurkat cells treated with fungal taxol or baccatin III revealed similar time- and concentration-dependent induction of apoptosis. However, increased apoptotic (hypoploid) sub G1 cells after fungal baccatin III treatment occurred at higher concentration compared to fungal taxol. This might be either due to the high affinity of taxol to microtubules [40] or involvement of non-tubulin factors [41]. Under the conditions used in this study, both fungal taxol and baccatin III induce apoptotic cell death in JR4-Jurkat, HepG2, HeLa, Ovcar3 and T47D cells with very similar kinetics, as determined by the appearance of hypoploid DNA, although the concentrations of fungal taxol varied from 10 nM for T47D, JR4-Jurkat and HeLa cells, to 100 nM for HepG2 and Ovcar3 cells. Half maximal concentrations of baccatin III in contrast, varied between a narrow range of 2–5 μM for all these cells. These results indicate that although taxol induces apoptosis in all the cells tested, there are sensitivity differences between the cell lines towards fungal taxol and baccatin III treatment.

Fungal taxol- and baccatin III- induced apoptosis was demonstrated by morphological criteria after staining with Hoechst or AO-EB staining. A significant loss in the mitochondrial membrane potential was obtained upon treatment of JR4-Jurkat cells with fungal taxol and baccatin III reaching up to 80% after 36 h at the given concentration. Convincing genetic evidence has been provided to show that taxol-mediated apoptosis solely relies on the mitochondrial pathway [15]. Baccatin III has been shown to induce apoptosis in human breast cancer (Bcap37) and epidermal carcinoma (KB) cell lines [40], but the mechanism is not fully understood. Furthermore, JR4-Jurkat and HeLa cells treated with 0.1 μM fungal taxol or 3.5 and 3 μM of baccatin III respectively showed a considerable increase in the percentage of apoptotic nuclei after 12 h incubation.

DNA fragmentation in a ladder-like fashion, one of the main hallmarks of apoptosis, was observed upon treatment of the cell lines with fungal taxol and baccatin III and it occurs at 6 nM (JR4-Jurkat cells) and (HeLa cells) fungal taxol, and 3.5 μM (JR4-Jurkat cells) - 3 μM (HeLa cells) fungal baccatin III.

The requirement of caspase-10 activation downstream of mitochondria in taxol-induced apoptosis has been reported earlier [13,18]. Earlier it was shown that caspase-10 is involved in etiposide-induced apoptosis in U937 human leukemic cell line [42] and flunarizine (Ca(2+) channel blocker)-induced apoptosis in Jurkat cells [43]. In this study, Specific involvement of caspase-10 has been demonstrated in apoptosis of JR4-Jurkat cells induced by fungal taxol and baccatin III, employing the inhibitors of caspase-9, -3, -2 and -10.

Baccatin III is known to be the precursor of taxol. But the experiments with respective growth over a period of time did not show the expected precursor-product relationship [44]. The presence of high concentration of baccatin III during the growth period may therefore indicate that this molecule by itself is active and may even have other roles. Further, the ester bond at C13 position of taxol is likely to be hydrolyzed during transport into the cell and thereby yield a higher intracellular concentration of baccatin III. Substantiating this hypothesis would explain the higher efficacy of taxol. These studies suggest to us that baccatin III is probably the main active molecule inside the cell and calls for investigation into its intracellular actions.

In conclusion, this is the first report on the elucidation of the apoptotic mechanism of fungal baccatin III in cancer cell lines. The question whether taxol is more active than baccatin III in the induction of apoptosis because the ester is readily transported in the cell remains to be answered. As fungal baccatin III was found to be less active than fungal taxol in the *in vitro* studies, it is possible that fungal taxol, apart from its microtubule binding kinetics and interactions with other proteins may have advantage over baccatin III particularly in the cellular uptake process.

## Competing interests

The authors declare that they have no competing interests.

## Authors’ contributions

BVSKC, CJB and AAK contributed to the design of the study, the analysis of the data, and drafted the manuscript. BVSKC executed the experiments. GCK and RS participated in FACScan analysis. All the authors have read and approved the final manuscript.
